# Carbon Nanoparticles Extracted from Date Palm Fronds for Fluorescence Bioimaging: In Vitro Study

**DOI:** 10.3390/jfb13040218

**Published:** 2022-11-04

**Authors:** Shaik Muhammad U. G. Mohiuddin, Abdu Saeed, Ahmed Alshahrie, Adnan Memić, Fadwa Aljoud, Shittu Abdullahi, Hussam A. Organji, Numan Salah

**Affiliations:** 1 Department of Physics, Faculty of Sciences, King Abdulaziz University, Jeddah 21589, Saudi Arabia; 2 Center of Nanotechnology, King Abdulaziz University, Jeddah 21589, Saudi Arabia; 3 Department of Physics, Thamar University, Thamar 87246, Yemen; 4 Department of Biological Science, Faculty of Science, King Abdulaziz University, Jeddah 21589, Saudi Arabia; 5 Regenerative Medicine Unit, King Fahad Medical Research Center, King Abdulaziz University, Jeddah 21589, Saudi Arabia; 6 Department of Physics, Faculty of Science, Gombe State University, Gombe 760253, Nigeria; 7 Center of Excellence in Desalination Technology, King Abdul-Aziz University, Jeddah 21589, Saudi Arabia

**Keywords:** date palm, carbon nanoparticles (CNPs), fluorescence, cell viability, bioimaging

## Abstract

Numerous studies have been reported on single- and multicolored highly fluorescent carbon nanoparticles (FCNPs) originating from various sources and their potential applications in bioimaging. Herein, multicolored biocompatible carbon nanoparticles (CNPs) unsheathed from date palm fronds were studied. The extracted CNPs were characterized via several microscopic and spectroscopic techniques. The results revealed that the CNPs were crystalline graphitic and hydrophilic in nature with sizes ranging from 4 to 20 nm. The unsheathed CNPs showed exemplary photoluminescent (PL) properties. They also emitted bright blue colors when exposed to ultraviolet (UV) light. Furthermore, in vitro cellular uptake and cell viability in the presence of CNPs were also investigated. The cell viability of human colon cancer (HCT-116) and breast adenocarcinoma (MCF-7) cell lines with aqueous CNPs at different concentrations was assessed by a cell metabolic activity assay (MTT) for 24 and 48 h incubations. The results were combined to generate dose-response curves for the CNPs and evaluate the severity of their toxicity. The CNPs showed adequate fluorescence with high cell viability for in vitro cell imaging. Under the laser-scanning confocal microscope, the CNPs with HCT-116 and MCF-7 cell lines showed multicolor fluorescence emissions, including blue, green, and red colors when excited at 405, 458, and 561 nm, respectively. These results prove that unsheathed CNPs from date palm fronds can be used in diverse biomedical applications because of their low cytotoxicity, adequate fluorescence, eco-friendly nature, and cheap production.

## 1. Introduction

Fluorescent carbon nanoparticles (FCNPs) have unique optical and chemical properties compared to previously used organic fluorescence probes. Carbon nanoparticles (CNPs) are emerging green nanoparticles with superior consonance emission capabilities compared to fluorescence semiconductors [1]. There are numerous methods for preparing FCNPs, including electrochemical and physical techniques, chemical vapor deposition, high-radiation-band preparation, thermal deposition, laser ablation, the oxidation of C-materials, and the chemical breakdown of fiber and carbohydrates [2,3,4,5].

Synthesized CNPs based on polymers, metals, silica, and semiconductors are known to possess better multiplexity abilities, making them excellent tools for biomedical applications, such as intracellular monitoring. However, their morphology and concentration remain their major drawbacks, leading to higher cytotoxicity effects. In this regard, it is necessary to investigate the balancing of organic CNP morphology and their doped chemical concentration to reduce their cytotoxicity through selectivity and sensitivity [6]. In addition, further studies are required to compare organic CNPs and traditionally available graphite and carbon nanotubes and fullerenes [7,8,9]. Retrospective studies [10,11] have reported that the highly improved soot-based synthesis of CNPs is straightforward, with a low quantum yield favorable in most biomedical applications.

The synthesis of CNPs from natural sources using green biosynthesis methods could be better for biomedical applications. The synthesis of CNPs based on trees as a carbon source as an alternative to chemical carbon sources could be environmentally and economically beneficial. Thus, in this work, we synthesized CNPs from date palm fronds. Millions of date palm trees are available worldwide. Date palm fronds are organic and represent a renewable resource, but these trees are burnt as waste for various reasons; therefore, utilizing them as a propitious bio-source for various biomedical applications would be beneficial. Furthermore, the biomass of date palm fronds was found to be rich in lignin content compared with the biomass of other fruits, making them an ideal biomass for carbon extraction. The biomasses extracted from date palm fronds are biocompatible with potential applications in bioimaging and theragnostics [12,13]. The biomass of date fruit is also nutrient-rich, containing high amounts of fibers and bioactive phytochemicals, exhibiting anticancer, anti-inflammatory, and antioxidant properties [10,14]. These properties, as well as cell viability, were investigated and validated by applying date extract to human colorectal cells (HCT-116) [10] and breast adenocarcinoma cell lines (MCF-7) [10,12]. CNPs extracted from date palm fronds could be a new green, facile, eco-friendly, and less expensive nanomaterial for bioimaging (carcinoma cells) and theragnostic applications.

In this report, novel CNPs with sizes of less than 10 nm were extracted from date palm fronds. The cell viability of the cell lines HCT-116 and MCF-7 in the presence of CNPs was also studied for potential applications in bioimaging. To the best of our knowledge, no study has reported the cell viability and dose-response curve under the application of high concentrations of CNPs to assess their biocompatibility. We also reported the bioimaging potential of crystalline graphitic CNPs in HCT-116 and MCF-7 cell lines through their fluorescence properties. Further investigations should explore the potential applications of these CNPs for cancer diagnosis, such as in multiphoton endomicroscopy [15], and treatment [15,16,17].

## 2. Materials and Methods

### 2.1. Materials

Date palm fronds (Phoenix dactylifera) were collected from date farms in Madinah, Saudi Arabia. Thiazolyl blue tetrazolium bromide (MTT) dye, trypsin-EDTA (0.25%), fetal bovine serum (FBS) growth media, phosphate-buffered saline (PBS) solution (pH 7.4), penicillin–streptomycin (Pen/Strep) antibiotic solution, dimethyl sulfoxide (DMSO) media solvent, and high-glucose Dulbecco’s Modified Eagle Medium (DMEM) containing phenol red and L-glutamine were obtained from Invitrogen (Waltham, MA, USA). SPL tissue culture flasks of sizes 75 and 25 cm^3^ with filter caps were purchased from SPL Fife-Sciences, Pocheaon, South Korea. CytoOne 96-well plates (Microtiter plates) with covering lids for cell culture were purchased from Scientific Incorporation, Ocala, FL, USA.

### 2.2. Synthesis of FCNPs

**Figure 1:**
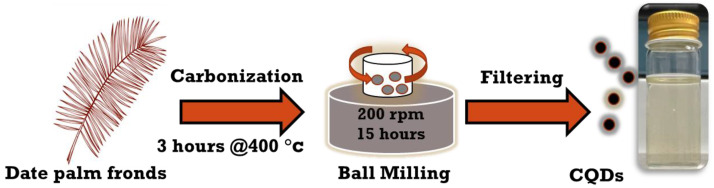
Schematic representation of top-down synthesis of CNPs.

As illustrated in Figure 1, CNPs were synthesized according to published works [18,19] by carbonizing the date palm fronds at 400 °C for approximately 3 h, followed by high-energy ball milling for 15 h with a rotating speed of 200 rpm; the metallic jar contained four metallic balls of equal weight. The obtained carbon soot weighed around 25 g. Next, carbon powder was poured into DI water and mixed with a magnetic stirrer for 24 h at 70 °C; then, larger carbon particles were allowed to settle down, and the solution was filtered. Finally, the CNP solution was centrifuged at 4000 rpm for 1 h to obtain aqueous hydrophilic CNPs. The concentration of the solution was 16.6 mg/mL, with a measured pH of 7.23. Different concentrations of aqueous CNPs were extensively prepared by diluting them with only DI water as a solvent to analyze the cell viability in response to concentrated CNPs. The synthesis of CNPs was performed at the Center of Nanotechnology, King Abdul Aziz University, Jeddah, Kingdom of Saudi Arabia.

### 2.3. Characterization of CNPs

The characterizations of CNPs were performed at the Center of Nanotechnology, Jeddah, Saudi Arabia. The topographies of CNPs were determined using a scanning electron microscope (SEM) model JSM-7600F (JEOL, Tokyo, Japan) and a high-resolution transmission electron microscope (HR-TEM) model JEM 2100F (JEOL, Japan). The XRD was carried out using an Ultima IV model (Rigaku, Japan) at room temperature in continuous mode. It emitted graphite-monochromatized Cu-Kα radiation with a wavelength of λ = 1.54 Å. The applied current was 40 mA, and the applied voltage was 40 kV. The scanning axis for XRD patterns at 2θ had an angular range of 10 to 80°, and the first step was 0.05°. FTIR was used to trace the functional group present in the CNPs by applying the attenuated total reflection (ATR) sampling technique using a Nicolet iS10 model (Thermo-Scientific, Waltham, MA, USA). To record the photoluminescence emissions of the CNPs we used a spectral fluorophotometer model RF-5301 (Shimadzu, Japan). Absorption spectra of the CNPs were recorded with an ultraviolet–visible spectroscope model Lamda 750 (PerkinElmer, Waltham, MA, USA). The zeta potential of the CNPs was measured to determine the charges on the CNPs and their colloidal nature with cells using a Malvern particle size analyzer model v2.3 (Malvern Instrument Ltd., Worcestershire, UK).

We estimated the carbonization by investigating the chemical elements using energy dispersive X-ray (EDX) spectroscopy, as shown in Figure S1 and Table S1, to compare between the amount of carbon before and after processing the fronds. The functional groups before and after processing the fronds can be found in Figure S2 and are listed in Table S2. The results of the quantum yield test are summarized in Table S3 (more details are included in the Supplementary Materials.

### 2.4. Cytocompatibility Assessment of CNPs and In Vitro Imaging

#### 2.4.1. Cell Culture

The malignant cells, namely the HCT-116 and MCF-7 cell lines, were obtained from the Regenerative Medicine Unit, King Fahad Medical Research Center, Jeddah, Saudi Arabia, where the biological experiments of this study were conducted. Originally, the Regenerative Medicine Unit purchased the cell lines from the American Type Culture Collection (ATCC, Manassas, VA, USA). Cell culture flasks of 75 cm^3^ with lids were used for culturing cell lines. To maintain the cell growth, 10% (*v*/*v*) FBS in DMEM, as well as 1% Pen-Strep solution, was added. The culture flask was incubated under a humidified atmosphere at 37 °C, maintaining 5% CO_2_. The culture media were replaced when cells attained a high confluence of 90%. Finally, cell harvesting was performed using trypsin solution.

#### 2.4.2. Cell Viability Assay (MTT Assay)

Following cell harvesting, the cells were seeded using microtiter plates containing ~5000 cells/well. These plates were supplemented with cell growth media and incubated for 24 h under the incubation conditions mentioned above, along with 95% air for adherence. Furthermore, CNPs were prepared at different concentrations (0.5, 1, 2.5, 5, 7.5, 10, 12.5, and 15 mg/mL), with a CNP concentration of 0 as the control. An MTT assay kit was used to estimate cell viability at time intervals of 24 and 48 h. The principal reaction of the MTT assay is based on the catalyzed conversion of soluble MTT tetrazolium into insoluble crystals of formazan, which are purple in color and produced by the succinate dehydrogenase within mitochondria. The MTT assay was conducted according to the manufacturer’s guidelines.

The MTT solution was prepared by dissolving 5 mg/mL of PBS in MTT powder; each well was supplemented with 10 µL of the prepared MTT solution, followed by a further 4 h of incubation at 37 °C with 5% CO_2_, maintaining a humid atmosphere. Later, 25 µL of media was removed from each well to dissolve formazan in 50 µL of DMSO [20], mixed with a pipette. Under the same incubation conditions, the cells were incubated for 10 min. Finally, using the microplate reader spectrometer in Synergy2 (Gen5 software, Bio-Tek, Winooski, VT, USA), the absorbance at 540 nm was recorded. The number of living cells corresponded to the absorbance value. Cell viability was calculated using the control wells and excluding blank wells. The percentage of control values was used to calculate the cell viability % as the absorbance value [21]. The cell viability % was calculated using the following equation [22]:

(1) $$\mathrm{CV}\%=\frac{A_{\mathrm{CQD}}{\;-\;A}_{\mathrm{blk}}}{A_{\mathrm{cntrl}}{\;-\;A}_{\mathrm{blk}}}\;\times 100$$

where CV% is cell viability percentage; A is the mean absorbance value of incubated cells; and CQD, blk, and cntrl are the applied concentrations of CQD, blank wells, and control wells, respectively.

#### 2.4.3. Dose-Response Curve

Data obtained from the cell viability test were applied to plot the dose-response curve for cell lines HCT116 and MCF7 using the logarithmic function; the curve was plotted using cell viability % values versus the different concentrations of aqueous CNPs applied. The estimation of the CNP concentration doses applied to the cell lines was presented in the dose-response curve. The curves for EC20, EC50, EC80, and EC90 showed that these concentrations of CNPs caused the cell viability count to be 20, 50, 80, and 90%, respectively. A non-linear four-parameter logistic (4PL) sigmoid curve (Hill equation) was used to fit the dose-response curve. The curve was fit using the equation provided below [23].

(2) $$y=A1+\frac{A2-A1}{{1+10}^{\left(\mathrm{logEC}50-x\right)p}}$$

where A1 and A2 are horizontal lines, with A1 representing the minimum and A2 the maximum cell viability. Through estimations based on the curve, the optimal concentration of aqueous CNPs was prepared to apply to imaging cells.

#### 2.4.4. Laser-Scanning Confocal Imaging

The in vitro imaging of cell lines HCT-116 and MCF-7 was carried out using a laser-scanning confocal microscope. The cell lines were cultured in a 25 cm^2^ cell flask containing media. The detaching process was performed by applying 0.25% trypsin EDTA when a high cell confluency was reached. Then, cells were suspended in DMEM-based media before collection onto the slide. Ten microliters of cells was dropped onto the slides and left for 2 h. Finally, the slides containing the cells were placed into a petri dish and incubated under the abovementioned conditions before imaging with and without aqueous CNPs. Images of cells were captured using a high-resolution scanning confocal laser microscope, model LSM 780, from Carl Zeiss, Aalen, Germany; the laser system was controlled digitally using ZEN 2010 software.

### 2.5. Statistical Analysis

Standard deviations (SDs) were taken as the average value of the input data. The significance of the statistical analysis was evaluated using the Student’s *t*-test, resulting in a value of *p* > 0.05. All statistical analyses were performed through Origin version 2022b (Northampton, MA, USA).

## 3. Results and Discussion

### 3.1. Physiochemical Characterization

**Figure 2:**
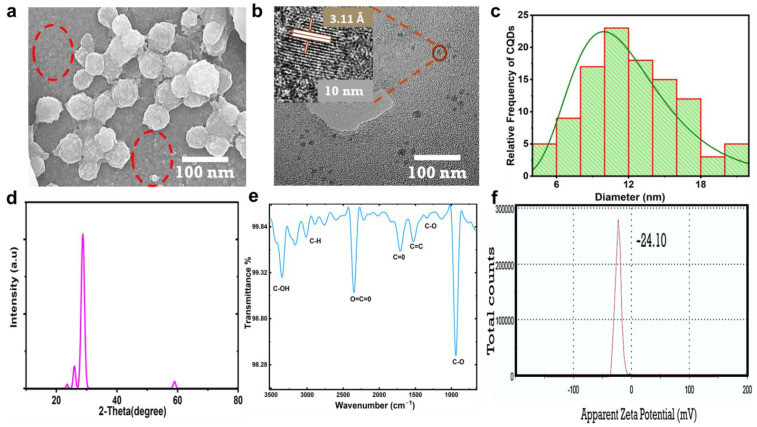
Morphology, structure, and physiochemical characterization of CNPs. (**a**) The CNPs were imaged using SEM at 100 nm magnification; small particles are highlighted within a red circle. (**b**) TEM image and HR-TEM at 10 nm displaying crystalline phase of CNPs and interlayer spacing of 3.51 Å. (**c**) Histogram plot of the measured sizes of CNPs. (**d**) XRD pattern. (**e**) FTIR. (**f**) Zeta potential distribution of CNPs, indicating surface charges and colloidal stability.

The CNPs showed homogeneous distribution; they appeared to agglomerate as balls, as shown in the SEM image in Figure 2a. Within the red circle, the CNPs seem to detach from one another rather than agglomerate; at the same time, the small CNPs gathered to form agglomeration balls, wherein the small CNPs appeared as lobes accumulated on the surface of the balls. The HR-TEM image presented in Figure 2b displays CNPs 10 nm in size with a crystalline nature and a d-spacing of 3.51 Å (002), which indicates a CNP crystalline graphitic sp2 phase [23,24]. Figure 2c shows the histogram plot for the size distribution of CNPs estimated using FIJI ImageJ software; the CNP sizes ranged from 4 to 20 nm. The HR-TEM in Figure S3 was used to estimate the average particle size, and 107 particles were counted. The X-ray diffraction (XRD) characteristic pattern was acquired to confirm the CNP crystallinity, as shown in Figure 2d. A sharp diffracted peak appeared at 28.75°, with a d-spacing of 3.11 Å (002) [24,25], which was attributed to the CNPs’ graphitic crystalline nature and bulk graphite with oxygen as the only functional group attached. The d-spacing of 3.44 Å (002) was in good agreement with the results recorded using HR-TEM; a small peak at 59° was observed with a d-spacing value of 1.56 Å (103), which was attributed to graphitic diffraction [26]. Figure 2e shows the CNP functional groups, confirming the excess oxygen, with the transmittance spectra recorded via Fourier transform infrared spectroscopy (FTIR) displaying bands at 1335, 1398, 1523, and 1700 cm^−1,^ which could be assigned to the stretching vibrations of C-O, C-O-C, C=C, and C=O [15,26,27,28,29], respectively. A strong band at 2350 cm^−1^ could be assigned to the O=C=O stretching vibrations; this band was reported as background carbon dioxide CO_2_ [30,31,32] or the possible entrapment of CO_2_ in porous CNPs [33]. The extensive bands from 2763 to 3163 cm^−1^ could be assigned to the stretching vibration of C-H, while the sharp band at 3400 cm^−1^ was attributed to vibrations of C-OH [33,34]. The zeta potential of the CNPs in Figure 2f showed that the surface of the CNPs was negatively charged, with a value of −24.10 mV; this revealed the repulsive colloidal nature of the CNPs in relation to each other [35], which was in good agreement with Figure 2a.

As was found in the results of the physiochemical properties of the CNPs presented in Figure 2a–f, the presence of carboxyl and hydroxyl functional groups could make CNPs highly hydrophilic in nature and play a major role in enhancing their fluorescence. Kavitha et al. [12] reported that the synthesis of mesoporous CNPs from date palms by carbonizing and piston grinding resulted in CNPs with an amorphous nature and the absence of fringes. On the other hand, Athinarayan et al. [13] reported crystalline CNPs synthesized from date palm using a hydrothermal process, which were found to have an inter-lattice spacing of 3.36 Å and a diffraction peak at 21.2° with a d-spacing of 3.34 Å, in contrast to the results of our study. Another study reported by Athinarayan et al. [36] indicated the presence of crystalline graphitic CNPs with an inter-lattice distance of 0.238 nm and a diffraction peak at 22.50° with a d-spacing of 0.356 nm, attributed to (100) carbon. Moreover, the CNPs synthesized in the above reports were found to have traces of nitrogen and a lower amount of oxygen present as functional groups. The CNPs obtained in this report were highly graphitic and crystalline, with diffraction peaks at 25.77° and 28.70° (002) and the presence of excess oxygen as the only surface functional group. Excess oxygen plays a vital role in creating defects within CNPs and increasing PL properties [37]. The absorption spectra reported in this study agreed with those in other reports [38,39,40].

### 3.2. Optical Characterization

**Figure 3:**
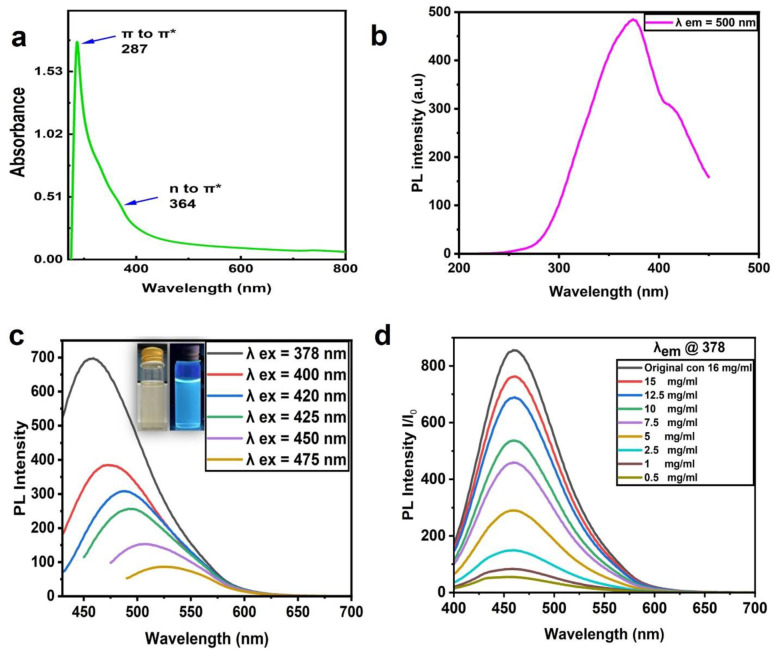
Absorption and PL properties of CNPs. (**a**) UV absorption spectra of CNPs. (**b**) PL excitation spectra at the emission wavelength of 378. (**c**) PL emission spectra at different excitation wavelengths. (**d**) PL emission spectra of aqueous CNPs at different concentrations (16 mg/mL (original), 0.5, 1, 2.5, 5, 7.5, 10, 12.5, and 15 mg/mL); all were excited at 378 nm.

Figure 3a shows the CNP absorption spectra with a sharp peak at 287 nm and a small shoulder at 364 nm. These absorption peaks were attributed to the electron transition levels of π -π* and n-π, revealing C=C and C=O functional groups, respectively [37,38]. The UV–visible absorption spectra of the CNPs were in good agreement with those of other reports [35,36]; Figure 3b demonstrates the excitation spectrum at the emission wavelength of 378. Figure 3c depicts the PL emission spectra of the CNPs at different excitation wavelengths, where the wavelength of 378 nm presented the maximum emission intensity at 464 nm. The emission intensity decreased with an increasing excitation wavelength, and the peaks shifted. The aqueous CNPs excited at 365 nm using UV light model: UVP-LLC, UVGL -58(Upland, Ca, USA) emitted a bright blue color, seen in the inset of Figure 3c, which demonstrated fluorescence emission for the prepared CNPs.

The CNP dye at a 16 mg/mL concentration diluted in deionized water (DI water) to different concentrations (0.5, 1, 2.5, 5, 7.5, 10, 12.5, and 15 mg/mL) is presented in Figure 3d. These CNPs were excited at 378 nm, and the highest emission intensity was observed at 16 mg/mL (the highest concentration) with an emission wavelength of 464 nm; otherwise, there was a decrease in intensity with increasing dilution.

### 3.3. Cell Viability and Dose-Response Curves

**Figure 4:**
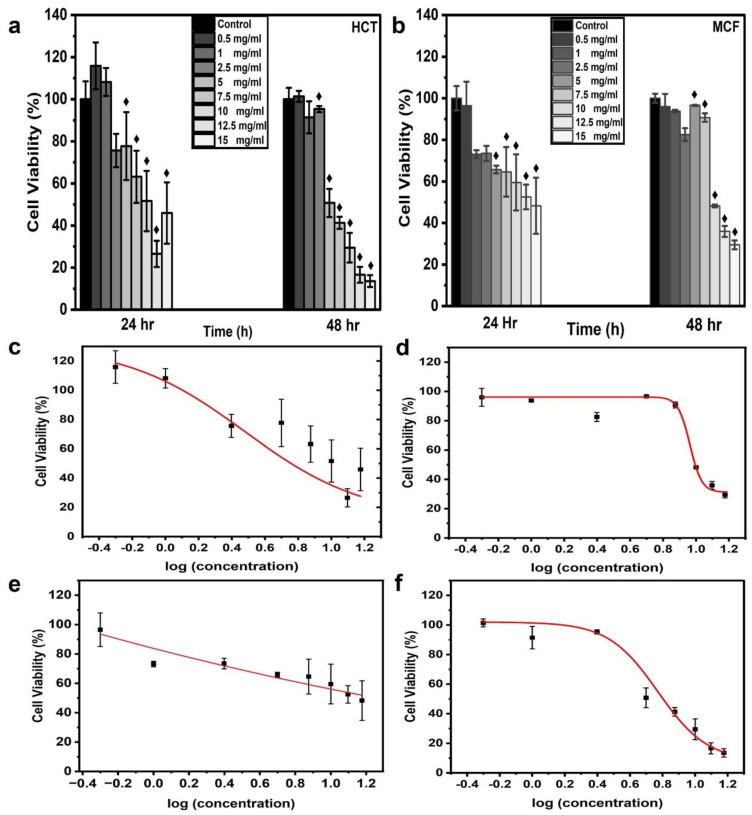
In vitro cytotoxicity and dose-response curve from CNPs. Histograms were plotted for MTT assay results to analyze cell viability upon the application of CNP concentrations of 0.5, 1, 2.5, 5, 7.5, 10, 12.5, and 15 mg/mL. Estimated cell viability % for cell lines (**a**) HCT116 and (**b**) MCF7, which were incubated for 28 and 48 h; ♦ indicates statistical significance at *p* < 0.05 as compared to the control value. The dose-response curves were generated for HCT-116 at (**c**) 24 h and (**d**) 48 h and for MCF-7 at (**e**) 24 h and (**f**) 48 h with the fitting equation; the curve was plotted as cell viability % (MTT assay) after incubation times of 24 and 48 h versus logarithmic concentrations of CNPs.

The cell viability of the aqueous CNPs was assessed for different concentrations (0.5, 1, 2.5, 5, 7.5, 10, 12.5, and 15 mg/mL) of CNPs. In this study, CNPs were applied to two famous carcinoma cell lines, namely HCT-116 and MCF-7. The CNPs were dispersed with MCF-7 and HCT-116 cell lines in a 96-well microtiter cell plate. Each cell line was cultured in three wells to obtain triplicate results, with each concentration applied. The average absorbance value of the three wells was used to determine the cell viability, which was calculated using Equation (1). Figure 4a,b show the frequency distribution of cell line viability at different applied concentrations of CNPs. At 0.5 mg/mL for 24 h of incubation, the maximum viability of the HCT-116 and MCF-7 cells was 115.86 and 96.47%, respectively. The viability of more than 100% might have been due to the proliferation of the HCT-116 cells, thereby resisting the applied dose of CNPs [41,42,43]. On the contrary, a viability greater than 100% was not observed at 48 h of incubation. The highest concentration of CNPs (15 mg/mL) resulted in a viability of 44 and 45.94% for HCT-116 and MCF-7 cell lines, respectively, which dropped to ~55% after 24 h of incubation. At 48 h of incubation, the highest cell viability was found to be 101.38 and 95.97% at a 0.5 mg/mL CNP concentration for HCT-116 and MCF-7 cells, respectively; these results were close to the results at 24 h. On the other hand, the viability at the highest CNP concentration of 15 mg/mL dropped to 13.58 and 29.51% for the HCT-116 and MCF-7 cell lines, respectively. The possible reason for the higher cell viability after the longer incubation period of 48 h is the proliferation of the cells, which became more resistant to the CNPs at 48 h of incubation; this meant that the resistance of the cells increased and the toxicity decreased [41]. The MTT assay demonstrated that higher concentrations of CNPs caused decreasing cell viability for both incubation times. Furthermore, the malignant colon cells showed more resistance to CNPs than the malignant breast cells. These results were in agreement with those reported elsewhere [43,44]. The 0.5-mg/mL concentration dose of CNPs used in the MTT assay was still much higher than what is required for cell imaging. The cell viability exceeded 100% at this concentration, making it an ideal CNP concentration for bioimaging applications. For cell imaging, further analysis was performed to determine the appropriate CNP concentration of the dye. The cell viability ratio was compared with the control cells in the presence of zero CNPs, presented as an average value with SD.

Dose-response curves were generated using the experimentally obtained values of cell viability % at 24 and 48 h of incubation versus the logarithmic concentrations of CNPs for both cell lines. The curves were fitted using the fitting equation. EC50 represents 50% cell viability in response to the applied concentration, so the sigmoid curve at EC50 was denoted as the Hill slope. The dose-response curves for the HCT-116 and MCf-7 cell lines demonstrated a decreasing order of cell viability with corresponding concentrations, as observed from the EC90, EC80 EC50, and EC20 values. The values for EC90, EC80, EC50, EC20, and R2 are summarized in Table 1. The fitted curve displayed R2 values equal to and greater than 0.9709. An MCF-7 cell line response curve for 24 h of incubation was not obtained due to the negligible cell viability changes, leading to a linear response between the applied concentrations [10]. The EC20, EC50, EC80, and EC90 cell viability values for the HCT-116 cell line were lower than those of MCF-7 for 48 h of incubation. MCF-7 cells indicated high cell viability in the presence of CNPs. The EC20 values of HCT-116 and MCF-7 were 9.61 ± 1.43 mg/mL and 10.29 ± 0.37 mg/mL, respectively. The EC50 values were 5.90 ± 0.55 mg/mL and 9.17 ± 0.34 mg/mL for HCT-116 and MCF-7, respectively. The EC80 and EC90 values were recorded as 3.63 ± 0.44 mg/mL and 8.18 ± 0.61 mg/mL and 2.73 ± 0.45 mg/mL and 7.64 ± 0.76 mg/mL, respectively. The EC20, EC50, EC80, and EC90 values for HCT-116 incubated for 24 h were 9.61 ± 1.43, 5.90 ± 0.55, 3.63 ± 0.44, and 2.73 ± 0.45, respectively.

Unsheathed aqueous CNPs from date palm fronds were evaluated for biocompatibility via an MTT colorimetric assay. Both carcinoma cell lines (HCT-116 and MCF-7) were incubated for 24 and 48 h with high CNP doses. The cell viability of the two cell lines was very high, with a survival rate greater than 95% at a 0.5 mg/mL concentration for both incubation times, as depicted in Figure 4. Furthermore, high cell viability was also observed for higher doses. The HCT-116 cell line survival rate remained above 100% at 24 h of incubation under a dose of 1 mg/mL, whereas the MCF-7 viability reduced to 70% and gradually started to decrease under high concentrations. Interestingly, both cell lines showed high cell viability (over 90%) at 48 h of incubation under concentration doses up to 2.5 mg/mL. Noticeably, the MCF-7 cell line showed excellent cell viability (more than 90%) at concentrations of up to 7.5 mg/mL. It can be concluded that at 48 h of incubation, both cell lines achieved excellent cell viability under high CNP concentrations. These results confirm that the CNPs extracted in this study had very low cytotoxicity and exemplary biocompatibility.

**Figure 5:**
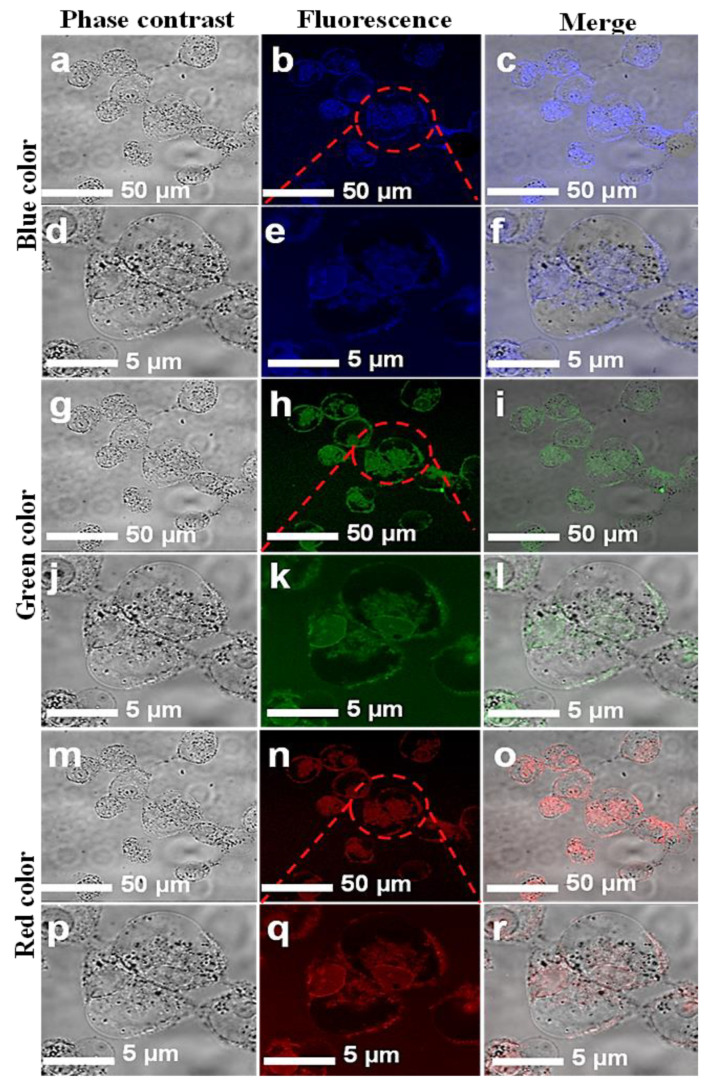
Confocal microscopic images of MCF-7 cell lines with introduced CNPs. Fluorescent confocal images of MCF-7 cells stained with CNPs at 0.5 mg/mL concentration. The images were captured at two different magnifications (50 and 5 µm). Images in the first column (**a**,**d**,**g**,**j**,**m**,**p**) are phase-contrast images; images in the second column (**b**,**e**,**h**,**k**,**n**,**q**) are fluorescence images; and images in the third column (**c**,**f**,**i**,**l**,**o**,**r**) are merged images of phase contrast and fluorescence.

**Figure 6:**
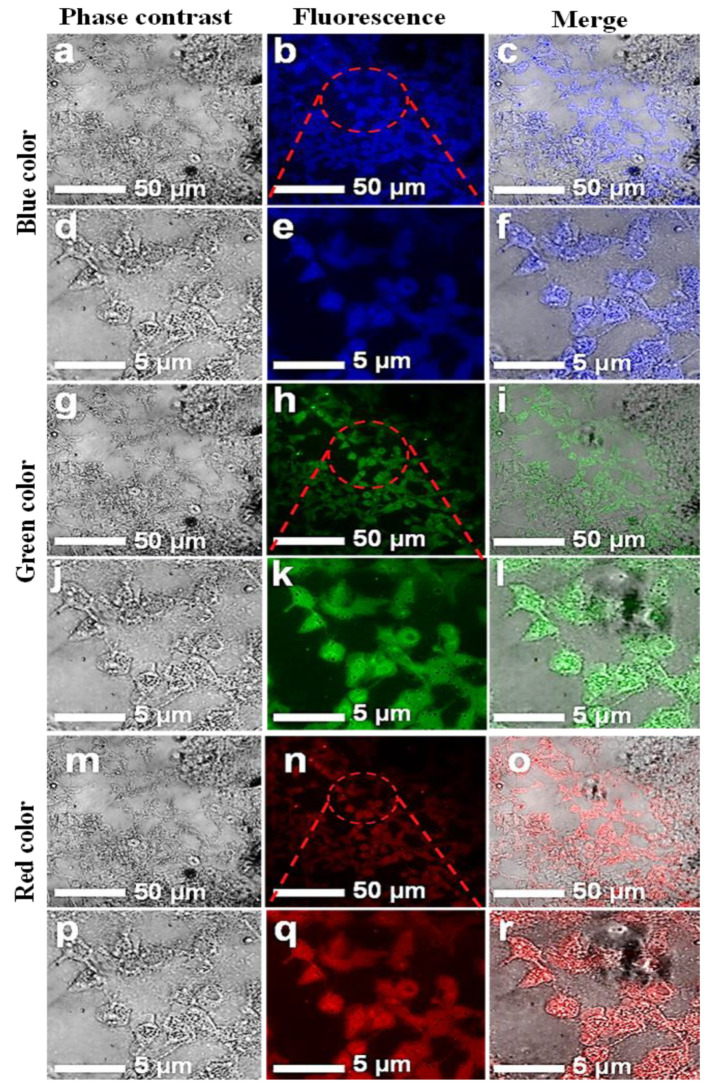
Confocal microscopic images of HCT-116 cell lines with introduced CNPs. Fluorescent confocal images of HCT-116 cells stained with CNPs at 0.5 mg/mL concentration. The images were captured at two different magnifications (50 and 5 µm). Images in the first column (**a**,**d**,**g**,**j**,**m**,**p**) are phase-contrast images; images in the second column (**b**,**e**,**h**,**k**,**n**,**q**) are fluorescence images; and images in the third column (**c**,**f**,**i**,**l**,**o**,**r**) are merged images of phase contrast and fluorescence.

The CNPs reported in this study contained oxygen as the only surface functional group. These CNPs were implemented for bioimaging using a laser-scanning microscope at wavelengths of 405, 458, and 561 nm and emitted mainly blue, green, and red colors, respectively, as depicted in Figure 5 and Figure 6. As seen in the FTIR results, the carboxyl and hydroxyl surface functional groups passivated on the CNPs may have played the predominant role in producing the efficient PL. The mechanism behind this may have been the trapping of excitons under excitation at certain wavelengths, followed by the repositioning of the trapped surface excitons and the recombination with radiative recombination centers [36]. It can be concluded from the above characterization results that the groups such as C=O can withdraw high numbers of electrons from the surface of the CNPs could lead to NIR emissions, causing a narrow band gap, irrespective of their carbonization content and the size of the CNPs. CNPs with high numbers of hydroxyl groups and low numbers of carboxyl groups have a weaker mechanism of electron withdrawal from the surface [17].

### 3.4. Cell Imaging by Laser-Scanning Confocal Microscope

The in vitro cellular uptake and the interaction of CNPs with individual HCT-116 and MCF-7 cells were imaged. A few drops of CNPs at a safer concentration dose of 0.5 mg/mL were applied to the cells. A laser-scanning microscope is used for cell imaging in DAPI mode. The samples were exposed at different excitation wavelengths, including 405, 458, and 561 nm. Figure 5 and Figure 6 show images of the cells while emitting multiple colors (blue, green, and red). These multicolor emissions were due to the independent-excitation PL emission property of CNPs [44,45,46]. Both cell lines took about 2 h for cellular uptake, as reported in previously published work [45]. The confocal images of both cell lines were taken at low and high magnifications, respectively. The magnified confocal images showed high fluorescence emission for CNPs taken up by endocytosis [35,46] and cell organelles. However, the localization of the individual cell organelles was not investigated. The MCF-7 cell image at a higher magnification revealed a fluorescent nucleus, which was apparently the largest part of the cell.

The images of both cell lines revealed that the CNPs have potential for bioimaging, since they had a zeta potential value of −24.10 (cationic) and a pH of 7.2, demonstrating their ability to penetrate into the cell membrane [47,48,49,50]. However, further investigations on the uptake of CNPs by individual cell organelles and nuclei are still required. The green emissions more effectively displayed the morphology of the cells compared with the other colors, and the cell organelles, such as the cell membrane, were visible due to fluorescence when excited under a confocal microscope. The extracted biocompatible CNPs with low cytotoxicity remained undistinguished in morphological appearance. Finally, in view of this investigation, the CNPs, without any functionalization, proved to have a high potential for biomedical applications such as bioimaging, biolabeling, biosensors, and drug delivery [35,50].

## 4. Conclusions

The CNPs investigated in this study are environment friendly and originate from date palm trees, with the potential to replace expensive toxic, metallic, and chemical fluorescent dyes for bioimaging. In this study, crystalline graphitic CNPs extracted from date palm fronds demonstrated exemplary PL properties at a pH of 7.23 for bioimaging. Investigations of the CNPs’ fluorescence lifetime, quantum yield at different wavelengths, and fluorescence mechanism need to be conducted in future studies. The immense bioimaging potential and biocompatibility of the CNPs were assessed by an MTT assay with two cell lines, HCT-116 and MCF-7. The adopted concentrations were high compared with those used in most published works due to CNPs’ organic nature. A lower toxicity was recorded under the effect of the CNP concentration of 0.5 mg/mL. The cell viability recorded at 24 h of incubation was 115.86 and 96.47% for HCT-116 and MCF-7 cell lines, respectively, while that at 48 h of incubation was 101.38 and 95.97%, respectively. Both cell lines showed higher cell viability at 48 h of incubation. Both the cell lines were stained with CNPs and then imaged via a fluorescence laser-scanning confocal microscope, which proved that the cell organelles were labeled. However, further studies on efficient strategies for the surface functionalization of CNPs are required for biolabeling, in vivo bioimaging, and various other biomedical applications.

## Figures and Tables

**Table 1:** Summary of the values of EC20, EC50, EC80, EC90, and R2 obtained from dose-response curves of HCT-116 and MCF-7 cell lines under CNP concentrations of 0.5, 1, 2.5, 5, 7.5, 10, 12.5, and 15 mg/mL.

Parameters	HCT		MCF	
	24 h	48 h	24 h	48 h
EC20 (mg/mL)	9.57 ± 16.11	9.61 ± 1.43	–	10.29 ± 0.37
EC50 (mg/mL)	3.01 ± 1.61	5.90 ± 0.55	–	9.17 ± 0.34
EC80 (mg/mL)	0.94 ± 1.07	3.63 ± 0.44	––	8.18 ± 0.61
EC90 (mg/mL)	0.48 ± 0.90	2.73 ± 0.45	–	7.64 ± 0.76
R^2^	0.970	0.993	–	0.993

## Data Availability

The data presented in this study are available upon request from the corresponding author.

## Supplementary Materials

The following supporting information can be downloaded at: https://www.mdpi.com/article/10.3390/jfb13040218/s1, Figure S1: EDX spectra for (a) raw date palm and (b) CNPs, showing the ire chemical elements; Figure S2: FTIR spectra of (a) extracted CNPs (b) Raw date palm fronds; Figure S3: HR-TEM image of CNPs used to estimate the average size of the particles; Table S1: Atomic percentages of chemical elements recorded by EDX for Raw date palm and extracted CNPs; Table S2: FTIR table assignment bands of Raw date palm fronds and their extracted CNPs; Table S3: Summarized parameters of CNPs for calculating the quantum yield compared to quinine sulfate. Ref. [51] in Supplementary Material.

## References

[1] Atabaev T.S. (2018). Doped Carbon Dots for Sensing and Bioimaging Applications: A Minireview. Nanomaterials.

[2] Campuzano S., Yáñez-Sedeño P., Pingarrón J.M. (2019). Carbon Dots and Graphene Quantum Dots in Electrochemical Biosensing. Nanomaterials.

[3] Khan Z.M.S.H., Saifi S., Shumaila, Aslam Z., Khan S.A., Zulfequar M. (2020). A Facile One Step Hydrothermal Synthesis of Carbon Quantum Dots for Label -Free Fluorescence Sensing Approach to Detect Picric Acid in Aqueous Solution. J. Photochem. Photobiol. A Chem..

[4] Zhao W.B., Liu K.K., Song S.Y., Zhou R., Shan C.X. (2019). Fluorescent Nano-Biomass Dots: Ultrasonic-Assisted Extraction and Their Application as Nanoprobe for FE3+ Detection. Nanoscale Res. Lett..

[5] Sharma A., Das J. (2019). Small Molecules Derived Carbon Dots: Synthesis and Applications in Sensing, Catalysis, Imaging, and Biomedicine. J. Nanobiotechnol..

[6] Bhunia S.K., Saha A., Maity A.R., Ray S.C., Jana N.R. (2013). Carbon Nanoparticle-Based Fluorescent Bioimaging Probes. Sci. Rep..

[7] Kong B., Tang J., Zhang Y., Jiang T., Gong X., Peng C., Wei J., Yang J., Wang Y., Wang X. (2016). Incorporation of Well-Dispersed Sub-5-Nm Graphitic Pencil Nanodots into Ordered Mesoporous Frameworks. Nat. Chem..

[8] Russo A.P., Hu G., Compagnini W.W., Duley N.Y.Z. (2014). Femtosecond Laser Large-Scale, Ablation of Highly Oriented Pyrolytic Graphite: A Green Route for Production of Porous Graphene and Graphene Quantum Dots. Nanoscale.

[9] Miao S., Liang K., Kong B. (2020). Förster Resonance Energy Transfer (FRET) Paired Carbon Dot-Based Complex Nanoprobes: Versatile Platforms for Sensing and Imaging Applications. Mater. Chem. Front..

[10] Soldevilla B., Díaz R., Silva J., Campos-Martín Y., Muñoz C., García V., García J.M., Peña C., Herrera M., Rodriguez M. (2011). Prognostic Impact of ΔTAp73 Isoform Levels and Their Target Genes in Colon Cancer Patients. Clin. Cancer Res..

[11] Qu S., Zhou D., Li D., Ji W., Jing P., Han D., Liu L., Zeng H., Shen D. (2016). Toward Efficient Orange Emissive Carbon Nanodots through Conjugated Sp2-Domain Controlling and Surface Charges Engineering. Adv. Mater..

[12] Kavitha T., Kumar S. (2018). Turning Date Palm Fronds into Biocompatible Mesoporous Fluorescent Carbon Dots. Sci. Rep..

[13] Athinarayanan J., Periasamy V.S., Alshatwi A.A. (2022). Unveiling the Biocompatible Properties of Date Palm Tree ( Phoenix Dactylifera L. ) Biomass-Derived Lignin Nanoparticles. ACS Omega.

[14] Rozila I., Abdul Manap N.M., Ghazali L.N., Kamal N., Abdul Hakeem W., Mohd Manzor N., Chowdhury S.R., Abdul Rahman S. (2019). The Antioxidant Properties and Anticancer Effect of Medjool Dates (Phoenix Dactylifera L.) on Human Breast Adenocarcinoma (MCF-7) Cells: In Vitro Study. Front. Pharmacol..

[15] Kučikas V., Werner M.P., Schmitz-Rode T., Louradour F., van Zandvoort M.A.M.J. (2021). Two-Photon Endoscopy: State of the Art and Perspectives. Mol. Imaging Biol..

[16] Zhang H., Wang G., Zhang Z., Lei J.H., Liu T.M., Xing G., Deng C.X., Tang Z., Qu S. (2022). One Step Synthesis of Efficient Red Emissive Carbon Dots and Their Bovine Serum Albumin Composites with Enhanced Multi-Photon Fluorescence for in Vivo Bioimaging. Light Sci. Appl..

[17] Bao X., Yuan Y., Chen J., Zhang B., Li D., Zhou D., Jing P., Xu G., Wang Y., Holá K. (2018). In Vivo Theranostics with Near-Infrared-Emitting Carbon Dots—Highly Efficient Photothermal Therapy Based on Passive Targeting after Intravenous Administration. Light Sci. Appl..

[18] Salah N.A.A., Salah Y.N.A., Alshahrie A.S.A. (2021). Methods of Producing Carbon Foreign Patent Documents Nanoparticles. U. Pat. U.

[19] Mohiuddin S.M.U.G., Aydarous A., Alshahrie A., Saeed A., Memić A., Abdullahi S., Salah N. (2022). Structural, Morphological, and Optical Properties of Carbon Nanoparticles Unsheathed from Date Palm Fronds. RSC Adv..

[20] Lei C., Cao Y., Hosseinpour S., Gao F., Liu J., Fu J., Staples R., Ivanovski S., Xu C. (2021). Hierarchical Dual-Porous Hydroxyapatite Doped Dendritic Mesoporous Silica Nanoparticles Based Scaffolds Promote Osteogenesis in Vitro and in Vivo. Nano Res..

[21] Scherließ R. (2011). The MTT Assay as Tool to Evaluate and Compare Excipient Toxicity in Vitro on Respiratory Epithelial Cells. Int. J. Pharm..

[22] Saeed A., Razvi M.A., Madkhli A.Y., Abdullahi S., Aljoud F., Zughaibi T.A., Aboushoushah S.F., Alshahrie A., Memic A., Al-Hazmi F.E. (2022). Investigation of the Tris(8-Hydroxyquinoline) Aluminum as a Promising Fluorescent Optical Material for in Vitro Bioimaging. Opt. Mater..

[23] Wang D.H., Li W., Liu X.F., Zhang J.M., Wang S.M. (2013). Chinese Medicine Formula “Jian-Pi-Zhi-Dong Decoction” Attenuates Tourette Syndrome via Downregulating the Expression of Dopamine Transporter in Mice. Evid.-Based Complement. Altern. Med..

[24] Sangam S., Gupta A., Shakeel A., Bhattacharya R., Sharma A.K., Suhag D., Chakrabarti S., Garg S.K., Chattopadhyay S., Basu B. (2018). Sustainable Synthesis of Single Crystalline Sulphur-Doped Graphene Quantum Dots for Bioimaging and Beyond. Green Chem..

[25] Li Y., Zhong X., Rider A.E., Furman S.A., Ostrikov K. (2014). Fast, Energy-Efficient Synthesis of Luminescent Carbon Quantum Dots. Green Chemistry.

[26] Larichev Y.V., Yeletsky P.M., Yakovlev V.A. (2015). Study of Silica Templates in the Rice Husk and the Carbon-Silica Nanocomposites Produced from Rice Husk. J. Phys. Chem. Solids.

[27] Döring A., Ushakova E., Rogach A.L. (2022). Chiral Carbon Dots: Synthesis, Optical Properties, and Emerging Applications. Light Sci. Appl..

[28] Zhu C., Zhai J., Dong S. (2012). Bifunctional Fluorescent Carbon Nanodots: Green Synthesis via Soy Milk and Application as Metal-Free Electrocatalysts for Oxygen Reduction. Chem. Commun..

[29] Alam A.M., Park B.Y., Ghouri Z.K., Park M., Kim H.Y. (2015). Synthesis of Carbon Quantum Dots from Cabbage with Down- and up-Conversion Photoluminescence Properties: Excellent Imaging Agent for Biomedical Applications. Green Chem..

[30] Wang B., Song A., Feng L., Ruan H., Li H., Dong S., Hao J. (2015). Tunable Amphiphilicity and Multifunctional Applications of Ionic-Liquid-Modified Carbon Quantum Dots. ACS Appl. Mater. Interfaces.

[31] Qian Z., Ma J., Shan X., Feng H., Shao L., Chen J. (2014). Highly Luminescent N-Doped Carbon Quantum Dots as an Effective Multifunctional Fluorescence Sensing Platform. Chemistry.

[32] Gerakines P.A., Schutte W.A., Greenberg J.M., van Dishoeck E.F. (1995). The Infrared Band Strengths of H2O, CO and CO_2_ in Laboratory Simulations of Astrophysical Ice Mixtures. Astron. Astrophys..

[33] Li Y., Wang K., Zhou W., Li Y., Vila R., Huang W., Wang H., Chen G., Wu G.H., Tsao Y. (2019). Cryo-EM Structures of Atomic Surfaces and Host-Guest Chemistry in Metal-Organic Frameworks. Matter.

[34] Gómez-Hernández R., Panecatl-Bernal Y., Méndez-Rojas M.Á. (2019). High Yield and Simple One-Step Production of Carbon Black Nanoparticles from Waste Tires. Heliyon.

[35] Qu D., Wang X., Bao Y., Sun Z. (2020). Recent Advance of Carbon Dots in Bio-Related Applications. J. Phys. Mater..

[36] Athinarayanan J., Periasamy V.S., Alatiah K.A., Alshatwi A.A. (2020). Synthesis and Cytocompatibility Analysis of Carbon Nanodots Derived from Palmyra Palm Leaf for Multicolor Imaging Applications. Sustain. Chem. Pharm..

[37] Das A., Kundelev E.V., Vedernikova A.A., Cherevkov S.A., Danilov D.V., Koroleva A.V., Zhizhin E.V., Tsypkin A.N., Litvin A.P., Baranov A.V. (2022). Revealing the Nature of Optical Activity in Carbon Dots Produced from Different Chiral Precursor Molecules. Light Sci. Appl..

[38] Wu Z.L., Liu Z.X., Yuan Y.H. (2017). Carbon Dots: Materials{,} Synthesis{,} Properties and Approaches to Long-Wavelength and Multicolor Emission. J. Mater. Chem. B.

[39] Siddique A.B., Pramanick A.K., Chatterjee S., Ray M. (2018). Amorphous Carbon Dots and Their Remarkable Ability to Detect 2,4,6-Trinitrophenol. Sci. Rep..

[40] Ganesan K., Ghosh S., Gopala Krishna N., Ilango S., Kamruddin M., Tyagi A.K. (2016). A Comparative Study on Defect Estimation Using XPS and Raman Spectroscopy in Few Layer Nanographitic Structures. Phys. Chem. Chem. Phys..

[41] Tang X., Wang L., Ye H., Zhao H., Zhao L. (2022). Biological Matrix-Derived Carbon Quantum Dots: Highly Selective Detection of Tetracyclines. J. Photochem. Photobiol. A Chem..

[42] Chen G., Qiu H., Prasad P.N., Chen X. (2014). Upconversion Nanoparticles: Design, Nanochemistry, and Applications in Theranostics. Chem. Rev..

[43] Yu X.F., Xiao B., Cheng J., Liu Z.B., Yang X., Li Q. (2019). Theoretical Design of Near-Infrared Fluorescent Sensor for F Anion Detection Based on 10-Hydroxybenzo[h]Quinoline Backbone. ACS Omega.

[44] Arya N., Arora A., Vasu K.S., Sood A.K., Katti D.S. (2013). Combination of Single Walled Carbon Nanotubes/Graphene Oxide with Paclitaxel: A Reactive Oxygen Species Mediated Synergism for Treatment of Lung Cancer. Nanoscale.

[45] Donahue N.D., Acar H., Wilhelm S. (2019). Concepts of Nanoparticle Cellular Uptake, Intracellular Trafficking, and Kinetics in Nanomedicine. Adv. Drug Deliv. Rev..

[46] Meng W., Bai X., Wang B., Liu Z., Lu S., Yang B. (2019). Biomass-Derived Carbon Dots and Their Applications. Energy Environ. Mater..

[47] Fan R.J., Sun Q., Zhang L., Zhang Y., Lu A.H. (2014). Photoluminescent Carbon Dots Directly Derived from Polyethylene Glycol and Their Application for Cellular Imaging. Carbon.

[48] Chang S., Chen B.B., Lv J., Fodjo E.K., Qian R.C., Li D.W. (2020). Label-Free Chlorine and Nitrogen-Doped Fluorescent Carbon Dots for Target Imaging of Lysosomes in Living Cells. Microchim. Acta.

[49] Qin J., Gao X., Chen Q., Liu H., Liu S., Hou J., Sun T. (2021). PH Sensing and Bioimaging Using Green Synthesized Carbon Dots from Black Fungus. RSC Adv..

[50] Kumar P., Dua S., Kaur R., Kumar M., Bhatt G. (2022). A Review on Advancements in Carbon Quantum Dots and Their Application in Photovoltaics. RSC Adv..

[51] Al-Abbad A., Al-Jamal M., Al-Elaiw Z., Al-Shreed F., Belaifa H. (2011). A Study on the Economic Feasibility of Date Palm Cultivation in the Al-Hassa Oasis of Saudi Arabia. J. Dev. Agric. Econ..

