# Supplementary material for: Carbon Nanoparticles Extracted from Date Palm Fronds for Fluorescence Bioimaging: In Vitro Study
Source: J Funct Biomater. 2022 Nov 4;13(4):218. doi: 10.3390/jfb13040218 (PMC9680435; doi:10.3390/jfb13040218)
Supplement: Supplementary file 1 [file jfb-13-00218-s001.zip › jfb-1981207-supplementary.pdf]

Supplement Information

Carbon Nanoparticles Extracted from Date Palm Fronds for Fluorescence Bioimaging: In Vitro Study

Shaik Muhammad U. G. Mohiuddin [1,2], Abdu Saeed [1,2,3], Ahmed Alshahrie [1,2], Adnan Memić [2], Fadwa Aljoud [4,5], Shittu Abdullahi [1,2,6], Hussam A. Organji [7] and Numan Salah [2,*]

[1] Department of Physics, Faculty of Sciences, King Abdulaziz University, Jeddah 21589, Saudi Arabia
[2] Center of Nanotechnology, King Abdulaziz University, Jeddah 21589, Saudi Arabia
[3] Department of Physics, Thamar University, Thamar 87246, Yemen
[4] Department of Biological Science, Faculty of Science, King Abdulaziz University, Jeddah 21589, Saudi Arabia
[5] Regenerative Medicine Unit, King Fahad Medical Research Center, King Abdulaziz University, Jeddah 21589, Saudi Arabia
[6] Department of Physics, Faculty of Science, Gombe State University, Gombe 760253, Nigeria
[7] Center of Excellence in Desalination Technology, King Abdul-Aziz University, Jeddah 21589, Saudi Arabia
* Correspondence: nsalah@kau.edu.sa

Energy Dispersive X-ray (EDX) analysis revealed the chemical element composition of raw date palm fronds and their extracted CNPs in Figure S1(a, b). Carbon, oxygen, and calcium were found in raw date palm fronds [51]. In CNPs, elements, namely carbon, and oxygen, were found. Silicon can be seen in the EDX due to the substrate used to analyze samples. Calcium disappeared in CNPs, possibly during carbonization, followed by the DI water-washing process. In Table S1, an increased atomic % of carbon content was noticed in CNPs compared to raw date palm fronds, with 25%

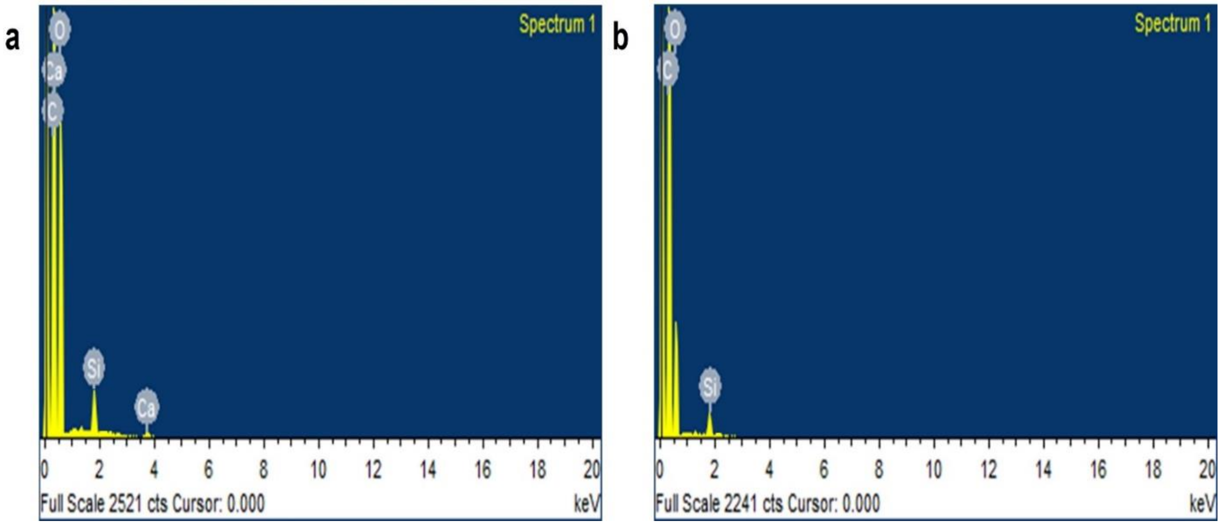

Figure S1. EDX spectra for (a) raw date palm and (b) CNPs, showing the ire chemical elements.

Table S1. Atomic percentages of chemical elements recorded by EDX for Raw date palm and extracted CNPs.

**Element**	**Raw date palm fronds (%)**	**Extracted CNPs (%)**
C K	55.78	80.37
O K	43.94	19.63
Ca K	0.28	–

FTIR characterization revealed the surface functional groups and stretching vibrations present in raw date palm fronds and extracted CNPs in Figure S2. Transmittance bands are summarized in Table S2 for functional groups. Compared to raw date palm fronds, the improvisation of carbon bands and carboxy functional groups was seen in CNPs. The abundant amount of carboxyl groups can be observed in the CNPs sample. A retrospective study proved that the presence of (C=O) electron-withdrawing groups on the surface could lead to emission near infrared [43].

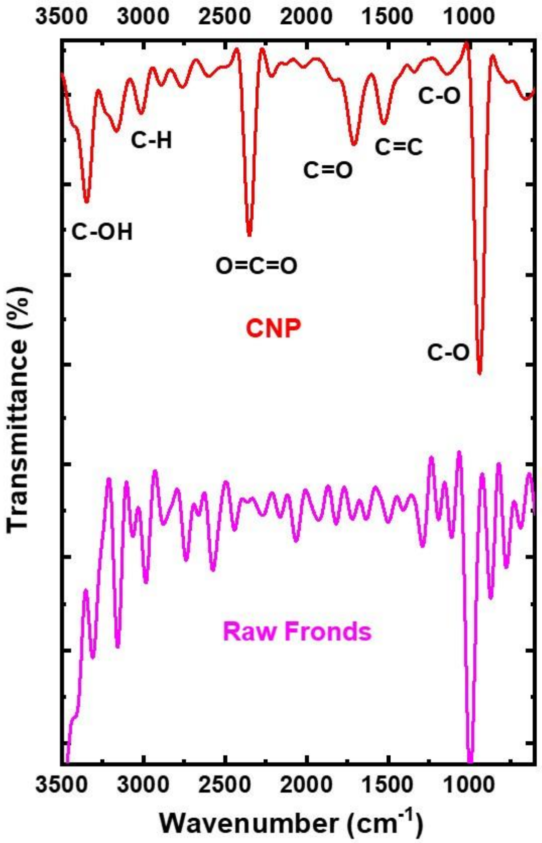

Figure S2. FTIR spectra of (a) extracted CNPs (b) Raw date palm fronds.

Table S2. FTIR table assignment bands of Raw date palm fronds and their extracted CNPs.

**Wavenumber (cm^{-1})**	**Band appearance**	**Functional group**	**Ref.**
1131	Stretching vibrations	C-O	[30,31]
1335	Stretching vibrations	C-O	[30,31]
1524	Small	C=C	[43,31]
1696	medium	C=O	[43,31]
2345	Stretching vibrations	O=C=O	[32,34]
2772	Stretching vibrations	C-H	[30,31]
2890	Stretching vibrations	C-H	[30,31]
3013	Stretching vibrations	C-H	[28,29,30]
3163	Stretching vibrations	C-H	[28,29,30]
3400	strong	C-OH	[28,29,30]

Table S3. Summarized parameters of CNPs for calculating the quantum yield compared to quinine sulfate.

Excitation wavelength (nm)	Refractive index (n)	The absorbance at 378 nm	Quantum yield
**378**	**1.33**	**0.399**	**3.24**

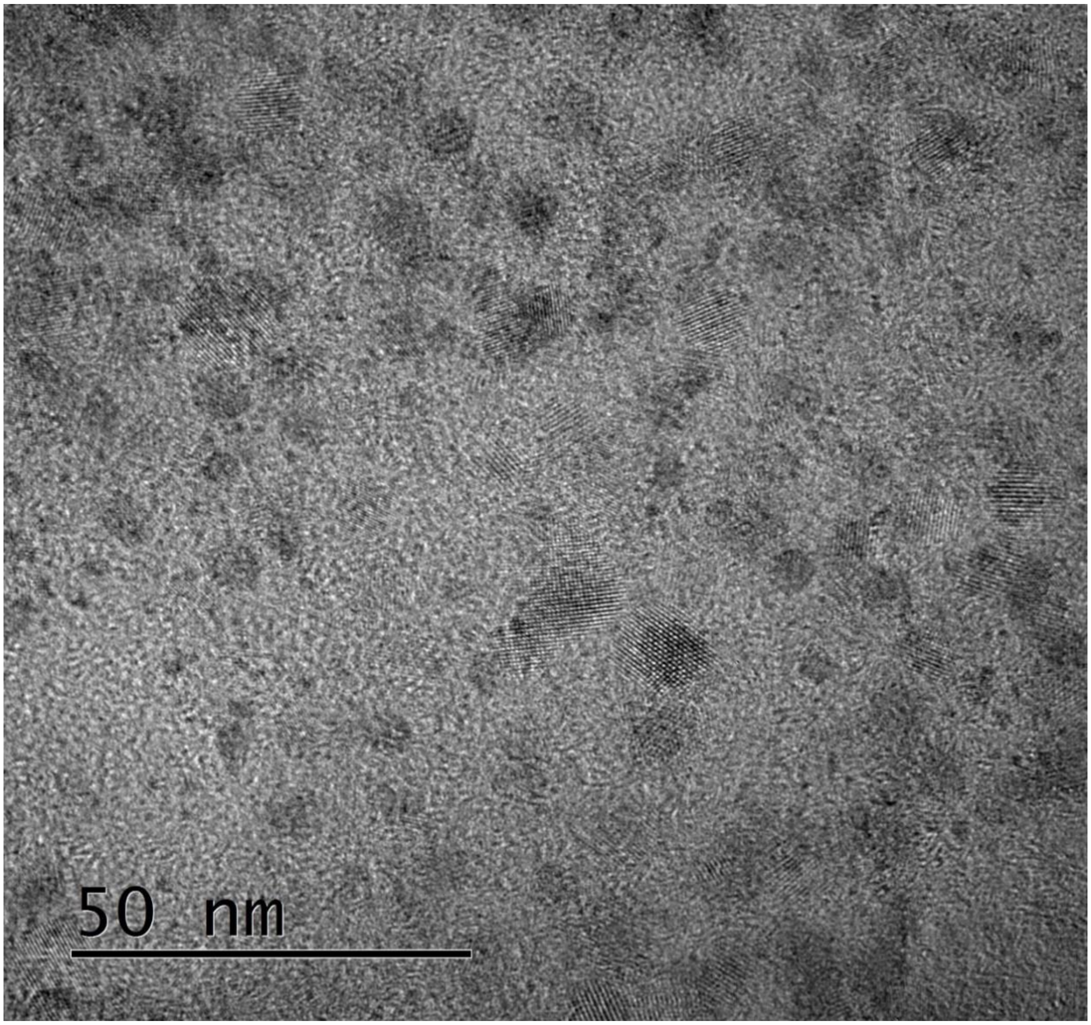

Figure S3 HR-TEM image of CNPs used to estimate the average size of the particles.
